# Long-term outcomes of accelerated partial breast irradiation with multicatheter interstitial brachytherapy versus whole breast irradiation: an 11-Year clinical practice follow-up study

**DOI:** 10.1007/s12094-025-04060-3

**Published:** 2025-09-28

**Authors:** Sara Garduño-Sánchez, María Isabel Villanego-Beltrán, Juan Gómez-Salgado, Javier Jaén-Olasolo

**Affiliations:** 1 Department of Radiation Oncology, Juan Ramón Jimenez University Hospital, Andalusian Health Service, Ronda Norte S/N, 21005 Huelva, Spain; 2 Department of Radiation Oncology, Puerta del Mar University Hospital, Andalusian Health Service, Ana de Viya 21 Avenue, 11009 Cadiz, Spain; 3 Department of Sociology, Social Work and Public Health, Faculty of Labour Sciences, University of Huelva, Avenida Tres de Marzo, S/N, 21007 Huelva, Spain; 4 Safety and Health Postgraduate Programme, Universidad Espíritu Santo, Av Samborondón 5, 092301 Guayaquil, Ecuador; 5 Biomedical Research and Innovation Institute of Cádiz (INiBICA) Research Unit, Puerta del Mar University Hospital, Andalusian Health Service, Ana de Viya 21 Avenue, 11009 Cadiz, Spain

**Keywords:** Breast cancer, Accelerated partial breast irradiation, Multicatheter interstitial brachytherapy, Clinical Oncology, Quality of Life, Late Toxicity, Cancer survival, Cosmetic results

## Abstract

**Purpose:**

To compare the long-term outcomes of accelerated partial breast irradiation (APBI) using multicatheter interstitial brachytherapy versus whole breast irradiation (WBI), in terms of late toxicity, cosmetic results, quality of life, and survival, in a real-world clinical setting.

**Methods:**

Updated analysis of two prospectively collected cohorts comprising 76 patients with stage I–II breast cancer treated with breast-conserving surgery followed by adjuvant radiotherapy. Patients who underwent APBI met the GEC-ESTRO eligibility criteria. We assessed follow-up and additional analyses of late toxicity, quality of life (assessed using the validated QLQ-BR23 and S-BIS questionnaires), cosmetic outcomes (via Visual Analog Scale), overall survival (OS), and disease-free survival (DFS), providing a more comprehensive evaluation of long-term outcomes.

**Results:**

At a median follow-up of 11 years, patient-reported quality of life remained significantly better in the APBI group, particularly in both physical and psychological domains, consistent with previous findings. No significant differences were observed in late clinical toxicity or cosmetic outcomes between groups. However, late mammographic findings showed a higher incidence of architectural distortion and tissue retraction in the APBI group, confirming earlier observations. The estimated 5- and 10-year OS rates were 94.7 and 81.1%, respectively. Corresponding DFS rates were 92.1% and 79.7%, with no statistically significant differences between treatment groups.

**Conclusion:**

With extended follow-up, our results reinforce that APBI using multicatheter interstitial brachytherapy is a safe and effective alternative to WBI in selected patients, providing long-term tumor control and survival comparable to WBI, while offering improved quality of life.

## Introduction

Breast-conserving therapy (BCT)—consisting of lumpectomy followed by radiotherapy—has demonstrated equivalent survival outcomes to mastectomy [1]. However, the prolonged duration of conventional radiotherapy regimens has posed practical challenges, especially for elderly patients and those with limited access to radiotherapy facilities [2].

Findings from pivotal trials, including the Canadian and UK studies (e.g., START-B) [3], have established hypofractionated whole breast irradiation (WBI) as a safe and effective alternative to historical conventional fractionation. More recently, ultrashort regimens such as FAST [4] and FAST-Forward [5], delivered in five fractions, have demonstrated non-inferior efficacy and are increasingly adopted in clinical practice.

The observation that most ipsilateral recurrences occur near the original tumor bed has led to the development of partial breast irradiation (PBI) techniques [6]. These include external beam radiotherapy (EBRT) using modalities such as 3D conformal radiotherapy (3D-CRT) [7], intensity-modulated radiotherapy (IMRT) [8], volumetric modulated arc therapy (VMAT) [9], and stereotactic body radiotherapy (SBRT) [10]; interstitial multicatheter brachytherapy [11] and balloon-based brachytherapy (e.g., MammoSite) [12]; as well as intraoperative radiotherapy (IORT) with electrons [13] or low-energy X-rays [14].

Within the broader category of PBI, accelerated partial breast irradiation (APBI) refers to a subset of techniques characterized by larger doses per fraction and shorter overall treatment durations, typically completed within one week [15]. While some protocols have employed conventionally fractionated EBRT, most APBI regimens use accelerated schedules delivered via either traditional or advanced technologies, such as 3D-CRT, IMRT, or brachytherapy [16].

Several randomized phase III trials have evaluated APBI as an alternative to WBI. The ELIOT and TARGIT-A trials tested IORT with electrons and low-energy photons, respectively [13, 14]. The Budapest trial [17] and NSABP B-39/RTOG 0413 [12] included both EBRT and brachytherapy arms. The GEC-ESTRO trial, however, uniquely compared APBI using multicatheter interstitial brachytherapy to standard WBI, employing both high-dose-rate (HDR) and pulsed-dose-rate (PDR) approaches [11].

Among the various APBI techniques, multicatheter interstitial brachytherapy offers superior dosimetric precision and effective sparing of organs at risk, making it a highly attractive modality for routine clinical use [15]. At least eight systematic reviews and meta-analyses have synthesized data from randomized trials, concluding that PBI provides overall survival outcomes comparable to WBI, with a more favorable toxicity profile [18]. However, results regarding local control have been somewhat heterogeneous. Outside the IORT setting, brachytherapy enables treatment over a shorter time frame and delivers lower radiation doses to critical organs, especially the skin, heart, and lungs [19].

In this context, we aimed to evaluate the long-term outcomes of APBI using multicatheter interstitial brachytherapy compared to WBI in routine clinical practice. This 2025 update provides extended follow-up of the cohort initially reported in 2019 [20], offering a comprehensive analysis of late toxicity, cosmetic outcomes, quality of life, local control, and survival.

## Materials and Methods

### Design

A comparative observational study was conducted involving two prospectively recorded cohorts of patients treated between 2008 and 2019, with follow-up data collected through 2025. The study included 76 women with early-stage breast cancer (stage I–II) who underwent breast-conserving surgery followed by adjuvant radiotherapy at Puerta del Mar University Hospital, Cádiz, Spain. In the analysis of the study endpoints, cosmetic assessment by physicians was not blinded to treatment allocation, as both clinical follow-up and treatment details were known.

### Ethics disclosure

All the procedures were carried out in accordance with the institutional care protocols and with the Declaration of Helsinki of 1964 and its subsequent modifications. The study was approved by the Research Ethics Committee of Huelva (Andalusia) (code: SICEIA-2024–002960). All participants gave informed written consent before being included and expressly agreed to participate in this study. The database used was retrospective with lack of patient intervention and all patient data were anonymized.

### Participants

A cohort of 37 patients received accelerated partial breast irradiation (APBI) via high-dose-rate (HDR) multicatheter brachytherapy with Iridium-192. All correspond to the low-risk category according to the GEC-ESTRO risk criteria for APBI: women > 50 years, unicentric/unifocal invasive tumors (pT1–2 pN0–1mic M0), no extensive intraductal or lymphovascular invasion, ≥ 2 mm margins, and no prior chemotherapy. Patients with prior breast irradiation or second tumors were excluded. The protocol used 8 × 4 Gy fractions. In 94.6% of cases, three treatment plans and ~ 15 catheters were used. Skin dose was capped at 2.7 Gy/fraction per GEC-ESTRO, ABS, and NSABP B-39/RTOG 0413 guidelines.

The comparator cohort comprised 39 patients treated with 3D-CRT whole breast irradiation (WBI) during the same period. They were randomly selected to match clinical and pathological features and met identical eligibility criteria. Treatment choice reflected practice variation and patient preference. Of these, 33.3% received hypofractionated WBI (40 Gy in 15 fractions), the most common regimen at our institution. All plans followed QUANTEC OAR dose constraints.

After completion of radiotherapy, patients were followed with a physical examination at 6 weeks, every 6 months during the first 3 years, and annually thereafter. Mammographic evaluations were performed 6 months after radiotherapy and then yearly.

### Outcomes

The primary outcomes included late toxicity, cosmetic outcomes, and quality of life. Late toxicity was evaluated according to the Common Terminology Criteria for Adverse Events (CTCAE) v4.0 and categorized by both clinical and radiological criteria. Clinical assessment included inspection for breast or scar tissue thickening, asymmetry, hyperpigmentation, and edema. Radiological toxicity was assessed using follow-up mammograms, focusing on findings such as fibrosis, architectural distortion, tissue retraction, and liponecrosis.

Quality of life was evaluated using the EORTC QLQ-BR23 [21] and the S-BIS (Body Image Scale) questionnaires [22]. Cosmetic outcomes were assessed using a 10-point visual analog scale (VAS), scored independently by patients and clinicians. Responses were analyzed individually and also grouped into physical, local physical, psychological, and sexual domains.

Secondary endpoints included overall survival (OS) and disease-free survival (DFS), estimated using the Kaplan–Meier method and compared with the log-rank test. OS was defined as the time from surgery to death from any cause or last follow-up, while DFS was defined as the time from surgery to any locoregional recurrence, second primary tumor or death.

In line with the definitions adopted by major clinical guidelines (NCCN, ESMO, SEOM), locoregional recurrence was defined as the appearance of disease within the locoregional territory of the treated breast, including the ipsilateral breast itself, defined as the reappearance of disease in the surgical scar or in the same quadrant, and the regional lymphatic drainage areas (axillary levels I–III, supraclavicular fossa, and internal mammary chain), occurring within the first 5 years after breast-conserving surgery. Distant metastasis was defined as the appearance of disease outside the locoregional territory of the treated breast whose histopathology was consistent with metastatic spread of breast cancer. Second primary tumors were defined as the occurrence of a new malignant tumor outside the locoregional territory of the treated breast. A second primary tumor in the same breast refers to a lesion in a different quadrant or located at a sufficient distance from the original surgical site, usually with a distinct histology. Even in cases where histology is the same, if the lesion appears more than 5 years after the first diagnosis, as many guidelines we consider it a new primary tumor rather than a recurrence.

### Procedures

Patients in the WBI group received 3D-conformal radiotherapy (3D-CRT) with 6 MV photons to the whole breast. Treatment used tangential field-in-field techniques and virtual wedges for dose homogeneity. Fractionation followed either conventional (2 Gy/fraction) or hypofractionated START-B (2.67 Gy/fraction) schedules. A tumor bed boost was given at the radiation oncologist’s discretion, based on institutional protocols and recurrence risk factors (e.g., age, tumor phenotype, size, lymphovascular invasion, DCIS).

Dosimetric data for the planning target volume (PTV) were derived from cumulative dose–volume histograms (DVHs), including V95 (the percentage of the PTV receiving ≥ 95% of the prescribed dose), and dose statistics such as D98, D2, and D50, representing the doses received by 98%, 2%, and 50% of the PTV, respectively. For organs at risk (OARs)—specifically the ipsilateral lung and, in left-sided tumors, the heart—analyses included mean organ doses, the volume of ipsilateral lung receiving ≥ 20 Gy (for conventional schedules) or ≥ 17 Gy (for hypofractionated schedules), and the heart volume receiving ≥ 30 Gy (conventional) or ≥ 28 Gy (hypofractionated).

In the APBI group, all patients received a total dose of 36 Gy, administered over one week as two fractions per day, in accordance with GEC-ESTRO guidelines [23, 24]. Prior to catheter implantation, patients underwent comprehensive imaging, including mammography, ultrasound, and magnetic resonance imaging (MRI). Additionally, targeted ultrasound was performed immediately before the procedure to locate the seroma, and computed tomography (CT) simulation was conducted with the templates in the planned position [15].

Implantation was performed under sedation using perforated templates to ensure optimal catheter geometry. CT-based treatment planning was conducted with radiopaque markers inserted into Elekta Comfort catheters, and dose calculations were performed using the Oncentra planning system. Dosimetric evaluation included planning target volume (PTV) coverage (V90%), Conformity Index (CI), implant volumes receiving ≥ 150% and ≥ 200% of the prescribed dose (V150 and V200), Dose Homogeneity Index (DHI), and Conformation Index (COIN), which accounts for both target coverage and implant geometry. The maximum skin dose was also recorded.

### Statistical analysis

Descriptive statistics were presented as means ± SD for continuous variables and as frequencies (%) for categorical ones. Chi-square or Fisher’s exact test assessed associations between categorical variables, as appropriate. Continuous variables were compared between groups using Student’s t-test.

Agreement on cosmetic outcomes (patient vs. clinician) was evaluated using the Intraclass Correlation Coefficient (ICC), and Pearson’s correlation assessed linear relationships between continuous variables.

Median follow-up was estimated via the reverse Kaplan–Meier method (deaths censored). Overall and disease-free survival (OS, DFS) were analyzed using Kaplan–Meier estimates and compared with the log-rank test.

Two-sided tests were used; p < 0.05 was considered significant. Analyses were conducted in SPSS v19.0 (SPSS Inc., Chicago, IL, USA).

## Results

### Characteristics of the study population

This analysis extends the previously published series with longer follow-up of the same patient cohort [20]. It includes 76 women (mean age: 66), with a median follow-up of 131.6 months (range: 18.7–247.3).

Baseline characteristics were similar between treatment groups, except APBI patients were older (mean age: 68.1 vs. 63.5; p = 0.01).

Most tumors were in the right breast (n = 45), mainly in the upper outer quadrant (n = 32). Invasive ductal carcinoma was predominant (n = 69); one case was DCIS. Grade 2 tumors (n = 39) and luminal A subtype (n = 58) were most common. Mean tumor size was 13 mm. Only four had pN1mic disease; the rest were pN0. Most were stage I (n = 67), with an average margin of 7 mm. Lumpectomy with SLNB was performed in 73 cases. Baseline characteristics of the study population are summarized in Table 1.

**Table 1:** Patient characteristics

	Total (n = 76)		WBI (n = 39)		APBI (n = 37)		Dif
	N	%	n	%	n	%	p-value
**Age**: mean (SD)	65.7 (7.6)		63.5 (7.0)		68.1 (7.6)		0.01
Histological type							0.27
Invasive ductal	69	90.8%	35	89.7%	34	91.9%	
Invasive lobular	2	2.6%	2	5.1%	0	0	
Ductal in situ	1	1.3%	1	2.6%	3	8.1%	
Other	4	5.3%	1	2.6%	0	0	
Histologic grade							0.07
G1	31	40.8%	11	28.2%	20	54.1%	
G2	39	51.3%	24	61.5%	15	40.5%	
G3	6	7.9%	4	10.3%	2	5.4%	
Phenotype							0.18
Luminal A	58	76.3%	27	69.2%	31	83.8%	
Luminal B	18	23.7%	12	30.8%	6	16.2%	
Axillary status							0.67
pN0	72	94.7%	37	94.9%	35	94.6%	
PN1mic	4	5.3%	2	5.1%	2	5.4%	
Staging							0.48
0 *(*in situ*)*	1	1.3%	1	2.6%	0	0	
I	67	88.2%	33	84.6%	34	91.9%	
II	8	10.5%	5	12.8%	3	8.1%	
Risk Group *(GEC-ESTRO criteria)*							
Low Risk	76	100%	39	100%	37	100%	
Type of surgery							0.11
Lumpectomy + SLNB	73	96.1%	39	100%	34	91.9%	
Lumpectomy + ALND	3	3.9%	0	0	3	8.1%	

*Dif*. Differences between WBI and APBI, *SD* Standard deviation, *SLNB* Sentinel lymph node biopsy, *ALND* Axillary lymph node dissection

Statistically significant differences in bold

### Treatment-related and dosimetric parameters

In the WBI group, the most commonly used protocol was hypofractionation similar to the START-B trial (40.05 Gy in 15 daily fractions over 3 weeks), the current standard fractionation per ESTRO guidelines, applied in 61.5% of patients. The remaining patients received historical conventional fractionation (50 Gy in 25 fractions over 5 weeks). A tumor bed boost was administered to 20 patients (51.3%), of whom 11 received it as part of the standard fractionation and 9 under historical conventional fractionation. Total doses ranged from 48 to 53 Gy in the standard fractionation and from 60 to 66 Gy in the historical conventional group. The mean V95% of the PTV was 98.4%. Mean lung doses were 7.4 Gy for historical conventional fractionation and 10.4 Gy for standard fractionation. In left-sided tumors, the average mean heart doses were 4.6 Gy (historical conventional fractionation) and 4.2 Gy (standard fractionation).

All patients in the APBI group received a total dose of 36 Gy delivered over one week (two fractions per day). In 94.6% of cases, three catheter planes were used, with an average of 16 catheters per implant. The mean PTV volume was 96.8 cm^3^. Dosimetric parameters—including V90%, Conformity Index (CI), Dose Homogeneity Index (DHI), and Conformation Number (COIN)—were consistently within recommended thresholds based on clinical guidelines [15, 25].

### Late side effects

No differences were observed between the groups in terms of late clinical toxicity, including parameters such as skin thickening, breast asymmetry, hyperpigmentation, or edema (Table 2).

**Table 2:** Late toxicity

	Total (n = 76)		WBI (n = 39)		APBI (n = 37)		Dif
	n	%	n	%	n	%	*p*-value
Late clinical toxicity							
Thickening of skin	66	86.8	32	82.1	33	89.1	0.31
Asymmetry	18	23.7	8	20.5	10	27.0	0.59
Hyperpigmentation	8	10.5	7	17.9	1	2.7	0.06
Edema	2	2.6	2	5.1	0	0	0.49
Late radiographic findings							
Fibrosis	57	75.0	30	76.9	28	75.7	0.90
Architectural distortion	45	59.2	14	35.9	31	83.8	< 0.001
Retractions	24	31.6	8	20.5	16	43.2	0.04
Liponecrosis	10	13.2	5	12.8	5	13.5	0.93
Cosmetic results (VAS)							
Patient-reported (*n* = 55) mean (SD)	8.4 (2.1)		7.9 (2.5)		8.8 (1.5)		0.10
Professional-reported (*n* = 35) mean (SD)	8.5 (1.4)		8.0 (2.0)		8.8(0.8)		0.10

*Statistically significant differences in bold*

*Dif*. differences between WBI and APBI, *CTCAE* v4.0 common terminology criteria for adverse event, version 4.0, *SD* standard deviation, *BI-RADS* breast imaging reporting and data system, VAS visual analogic scale

Late mammographic findings revealed a significantly higher incidence of architectural distortion and tissue retraction in patients treated with APBI compared to those who received WBI (83.8% vs. 35.9%, p < 0.001; and 20.5% vs. 43.2%, p = 0.04, respectively), confirming previous observations. No significant differences were found between the groups in the presence of fibrosis or liponecrosis.

The concordance between aesthetic outcomes reported by patients and those assessed by physicians was evaluated using the Intraclass Correlation Coefficient (ICC) for single measures. The resulting ICC was 0.70 (95% CI: 0.49–0.84), indicating a moderate to good level of agreement.

A significant correlation was found between physician-reported cosmetic outcomes in 2019 and 2025 (Pearson’s coefficient = 0.65; p = 0.03). In contrast, no statistically significant correlation was observed in patient-reported scores (Pearson’s coefficient = 0.14; p = 0.44). The mean VAS scores were 8.4 (patients) and 8.5 (physicians).

The comparison of late toxicity and cosmetic outcomes between 2019 and 2025, based on updated mammographic results and clinical evaluations by healthcare professionals, revealed no statistically significant changes, suggesting overall stability in these parameters over time.

### Quality of life assessment

After including additional patients who completed the quality-of-life questionnaires (six in the APBI group and seven in the WBI group), results remained consistent with those reported in the 2019 analysis, which included 25 patients per group.

Table 3 presents findings from a subgroup of 63 patients (31 in the APBI group and 32 in the WBI group) who participated in a patient-reported outcomes substudy using the EORTC QLQ-BR23 quality-of-life questionnaire and the S-BIS (Body Image Scale) Table 4).

**Table 3:** Quality of life questionnaires

EORTC QLQ-BR23	Total (n = 63)	WBI (n = 32)	APBI (n = 31)	Differences
	Mean (SD)	Mean (SD)	Mean (SD)	*p* value (95% CI)
General physical domain	1.4 (0.5)	1.5 (0.5)	1.2 (0.3)	< 0.01 [0.17–0.61]
Dry mouth	1.3 (0.6)	1.4 (0.7)	1.2 (0.5)	0.22 [−0.14 – 0.57]
Different flavour	1.2 (0.5)	1.4 (0.7)	1.0 (0.2)	0.04 [0.03–0.64]
Discomfort in eyes	1.2 (0.6)	1.2 (0.7)	1.3 (0.5)	0.68 [−0.42 – 0.27]
Hair loss	1.2 (0.4)	1.2 (0.5)	1.2 (0.4)	0.71 [−0.21 – 0.30]
Sensation disease	1.8 (1.2)	2.3 (1.3)	1.4 (1.0)	0.02 [0.16–1.46]
Blush/heat	1.6 (0.9)	1.8 (1.0)	1.3 (0.8)	0.05 [0.00–1.03]
Headaches	1.3 (1.3)	1.4 (0.9)	1.2 (0.5)	0.32 [−0.22 – 0.65]
Local physical domain	1.6 (0.6)	1.8 (0.6)	1.4 (0.5)	0.01 [−0.09 – 0.64]
Arm/shoulder pain	1.69 (0.9)	2.0 (0.9)	1.4 (0.7)	< 0.01 [0.22–1.15]
Swelling MS	1.43 (0.7)	1.6 (0.9)	1.2 (0.6)	0.07 [−0.03 – 0.80]
Difficulty raising MS	1.69 (0.8)	1.9 (0.9)	1.5 (0.8)	0.07 [−0.04 – 0.91]
Pain in the chest affected	1.86 (0.9)	2.1 (0.8)	1.6 (0.9)	0.07 [−0.04 – 0.93]
Chest swelling	1.63 (0.9)	1.8 (1.0)	1.4 (0.8)	0.13 [−0.12 – 0.91]
Breast sensitivity	1.76 (0.9)	2.0 (0.9)	1.5 (0.8)	0.06 [−0.01 – 0.97]
Skin involvement	1.59 (0.8)	2.0 (0.9)	1.2 (0.4)	< 0.01 [0.47–1.29]
Sexual domain	1.3 (0.4)	1.3 (0.4)	1.3 (0.4)	0.92 [−0.21 – 0.24]
Interest in sex	1.5 (0.6)	1.5 (0.7)	1.4 (0.5)	0.31 [−0.18 – 0.54]
Active sexual life	1.5 (0.7)	1.6 (0.8)	1.3 (0.6)	0.18 [−0.13 – 0.65]
Enjoy sex	1.3 (0.6)	1.4 (0.6)	1.2 (0.4)	0.39 [−0.18 – 0.45]
Psychological domain	1.6 (0.7)	1.6 (0.7)	1.4 (0.4)	< 0.01 [0.25–0.87]
Less attractive	1.6 ()	2.0 (1.1)	1.2 (0.6)	< 0.01 [0.26–1.27]
Less feminine	1.5 ()	1.7 (1.1)	1.2 (0.6)	0.05 [0.00–1.02]
Difficulty seeing your nudity	1.53 ()	1.8 (1.2)	1.2 (0.6)	0.03 [0.05- −1.14]
Body disillusion	1.4 ()	1.8 (1.0)	1.1 (0.3)	< 0.01 [0.18–1.08]
Concern about the future	2.6 ()	3.1 (1.1)	2.1 (1.0)	< 0.01 [0.42- −1.59]

Statistically significant differences in bold

*SD* standard deviation, *CI* confidence interval of the differences

**Table 4:** Spanish version of the body image scale (S-BIS)

S-BIS	Total (n = 63)	WBI (n = 32)	APBI (n = 31)	Differences
	Mean (SD)	Mean (SD)	Mean (SD)	*p* value (95% CI)
Body Scale Image	1.4 (0.7)	1.6 (0.9)	1.2 (0.9)	0.01 [0.10–0.76]
Self-conscious	1.8 (0.9)	2.1 (0.9)	1.4 (0.7)	< 0.01 [0.25–1.20]
Less physically attractive	1.5 (0.9)	1.9 (1.1)	1.2 (0.5)	< 0.01 [0.22- −1.22]
Dissatisfied wit appearance	1.4 (0.8)	1.7 (1.0)	1.2 (0.5)	0.05 [0.00–0.93]
Less feminine	1.3 (0.8)	1.6 (1.0)	1.0 (0.2)	0.02 [0.11- −0.98]
Difficult to see self-naked	1.4 (0.8)	1.7 (1.0)	1.1 (0.3)	0.02 [0.10–0.99]
Less sexually attractive	1.4 (0.8)	1.7 (1.0)	1.1 (0.3)	< 0.01 [0.19–1.06]
Avoid people	1.4 (0.8)	1.7 (1.0)	1.1 (0.3)	0.02 [0.09–1.01]
Body less whole	1.4 (0.8)	1.8 (1.0)	1.1 (0.3	< 0.01 [0.22–1.12]
Dissatisfied with body	1.4 (0.8)	1.8 (1.0)	1.2 (0.6)	0.02 [0.12–1.06]
Dissatisfied with scar	1.5 (0.8)	1.8 (1.0)	1.2 (0.6)	0.02 [0.12–1.06]

*Statistically significant differences in bold*

*SD* standard deviation, *CI* confidence interval of the differences

The APBI group demonstrated significant advantages in local physical, psychological, and body image domains. Relevant items included skin discomfort, dissatisfaction with surgical scars, perceived femininity, self-consciousness, and body image while undressed. Statistically significant differences favouring the APBI group were found in the general physical domain (mean score: 1.2 vs. 1.5, p < 0.01), local physical domain (1.4 vs. 1.8, p = 0.01), and psychological domain (1.4 vs. 1.6, p < 0.01). No difference was observed in the sexual functioning domain (1.3 vs. 1.3, p = 0.92), consistent with 2019 findings. Item-by-item analysis showed that all statistically significant differences favoured the APBI group.

In addition, the S-BIS results demonstrated significantly better outcomes for the APBI group across all 10 items, with a mean score of 1.2 compared to 1.6 in the WBI group (p = 0.01).

### Overall and disease-free survival

No locoregional recurrences occurred through 2025. Four patients developed distant metastases—two in each group. Both APBI patients also had metachronous second primaries: one parotid malignancy and one contralateral triple-negative breast cancer. In the WBI group, one had bone metastases, and another developed a second primary lung cancer.

Sixteen patients (9 WBI, 7 APBI) developed unrelated second malignancies, including lung (2 cases), colorectal (2), and isolated cases of liver, tongue, pancreatic, hematologic, bladder, and melanoma cancers. Three developed contralateral breast cancer after 8, 12, and 0 years (synchronous); all had distinct tumor profiles.

**Fig. 1:**
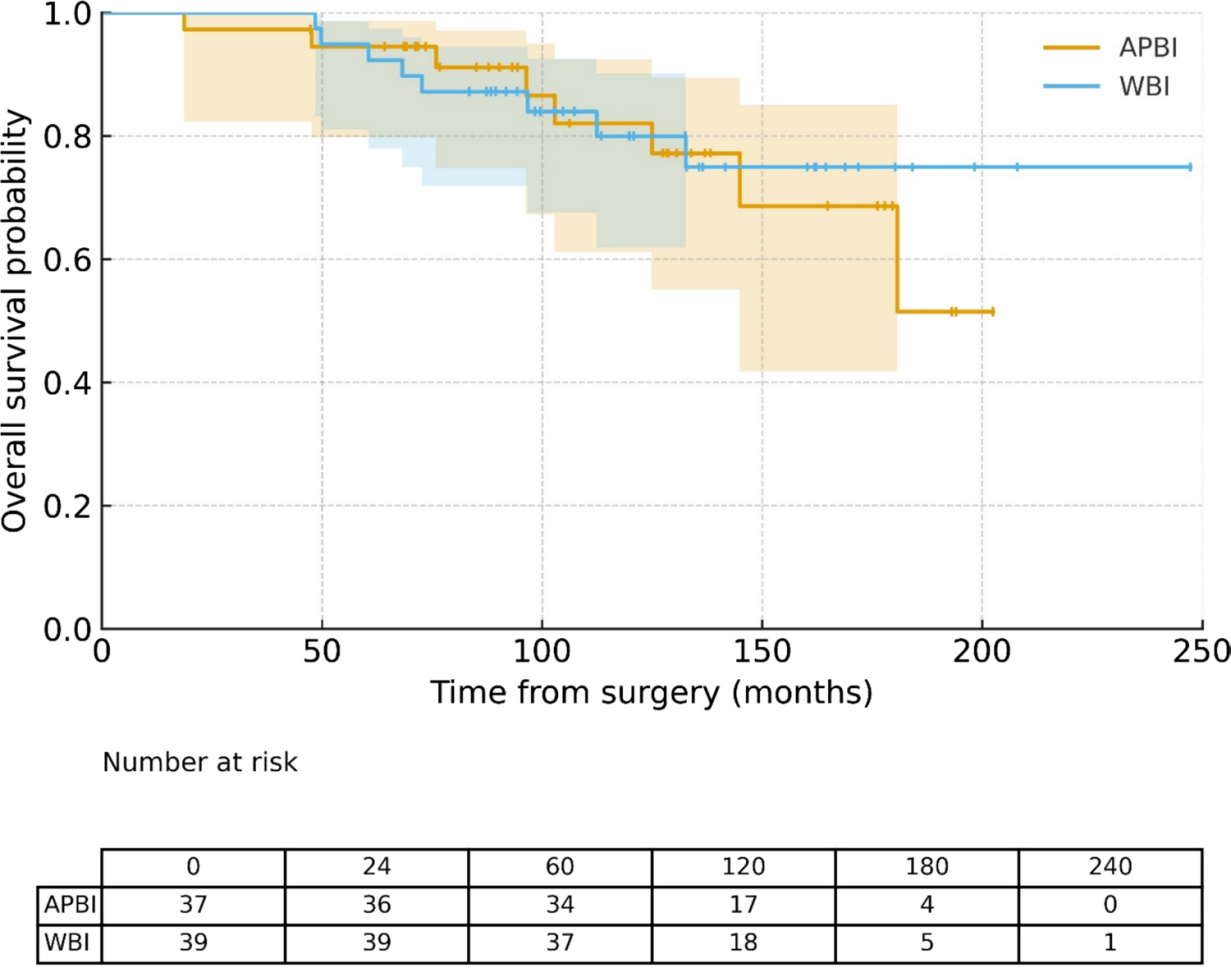
Overall survival curves by groups

**Fig. 2:**
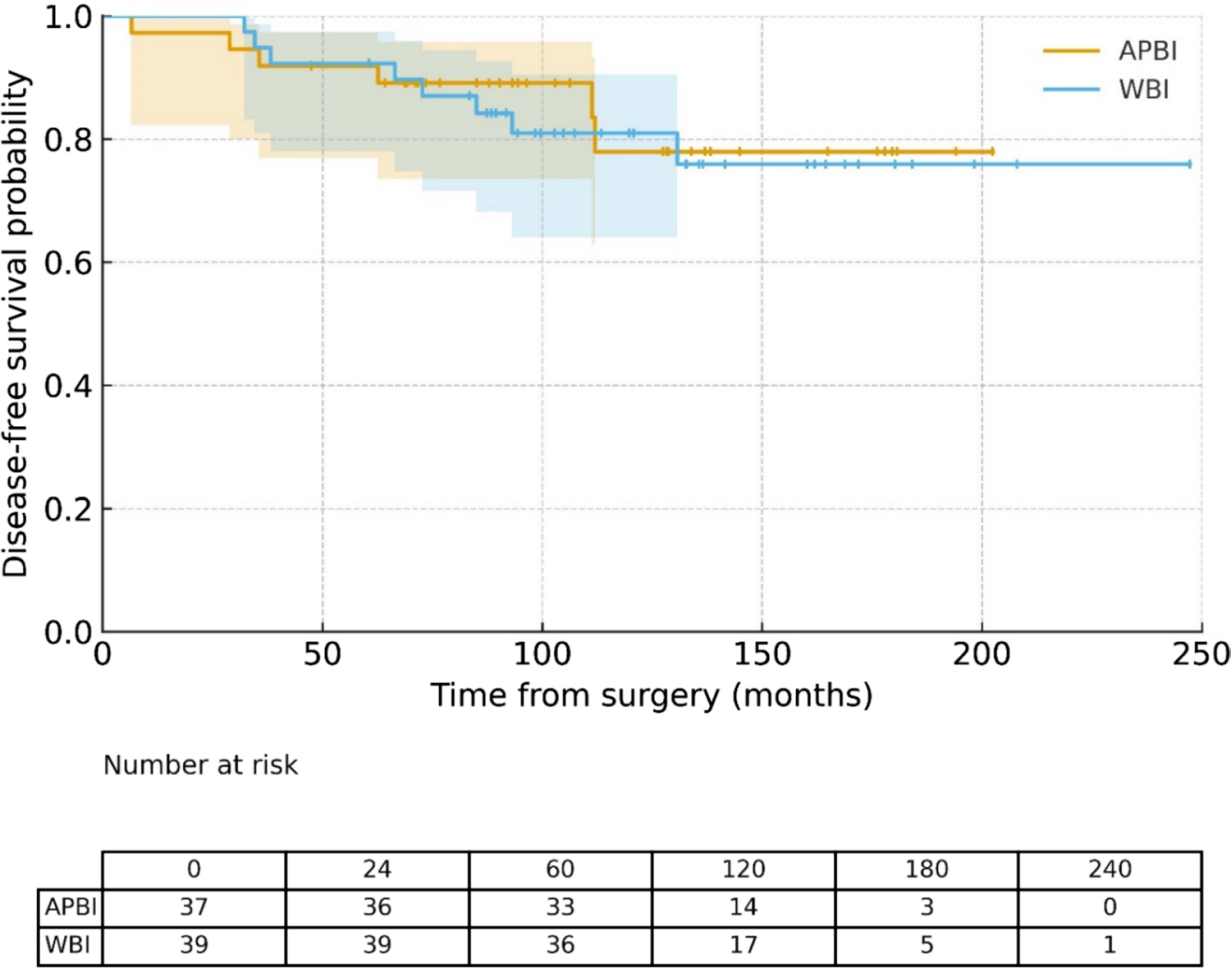
Disease-Free Survival curves by groups

The estimated 5- and 10-year overall survival (OS) rates were 94.7% and 81.1%, respectively (Fig. 1), while disease-free survival (DFS) rates were 92.1% and 79.7%, respectively (Fig. 2). At the time of analysis, neither the median OS nor the median DFS had been reached.

No statistically significant differences were observed between the APBI and WBI groups in terms of OS (p = 0.75) or DFS (p = 0.80), as shown in Figs. 1 and 2. Furthermore, no significant differences in OS or DFS were associated with patient age, the only baseline variable that differed between the groups.

## Discussion

There is robust evidence supporting the use of accelerated partial breast irradiation (APBI) beyond the confines of clinical trials. Both American (ABS [25], ASTRO [26]) and European (ESTRO [15]) radiation oncology societies have issued clinical recommendations endorsing its use. In this study, we compared APBI delivered via high-dose-rate (HDR) multicatheter interstitial brachytherapy (n = 37) with whole breast irradiation (WBI) (n = 39) in a routine clinical setting.

To date, only two randomized trials have directly compared WBI and APBI using multicatheter brachytherapy: the GEC-ESTRO and Budapest trials [11]. The NSABP B-39/RTOG 0413 trial also evaluated APBI, although only 6% of patients received multicatheter brachytherapy, with the majority treated using 3D-CRT (73%) or MammoSite intracavitary brachytherapy (21%) [12].

The GEC-ESTRO phase III non-inferiority trial enrolled 1,148 patients across multiple European centers, randomized to receive WBI or APBI using either HDR or pulsed-dose-rate (PDR) brachytherapy [11]. After a median follow-up of 6.6 years, the APBI group showed significantly lower rates of acute and late toxicity. Quality of life scores, assessed using the EORTC QLQ-C30 and QLQ-BR23, favored the APBI arm, particularly for breast symptoms and functional status. Cosmetic outcomes were also better with APBI, based on both physician and patient evaluations. Further analyses in 2017 and 2018 confirmed a reduced incidence of late skin toxicity and better quality of life.

The 2023 update reinforced these findings, confirming sustained oncologic efficacy and tolerability after more than 10 years of follow-up [27]. In our study, after a median follow-up of 11 years, no significant differences in overall survival or disease-free survival were observed between APBI and WBI, and quality-of-life and cosmetic outcomes were consistently superior in the APBI group. These findings are highly consistent with the recently published 10-year update of the GEC-ESTRO randomized trial [27], which represents the largest phase III study comparing multicatheter APBI and WBI. In that trial, more than 1,200 patients were randomized, and the 10-year ipsilateral local recurrence rate was 3.51% in the APBI arm versus 1.58% in the WBI arm, with a non-significant absolute difference of 1.93% (p = 0.074), well within the predefined non-inferiority margin. Importantly, the GEC-ESTRO trial confirmed that APBI was associated with significantly fewer grade ≥ 3 late side effects compared to WBI (1% vs 4%, respectively), with no grade 4 toxicities or treatment-related deaths.

When compared with our present results, both studies demonstrate long-term oncologic safety and tolerability of multicatheter APBI. While the GEC-ESTRO trial provides high-level evidence from a randomized setting, our prospective observational cohort confirms, in routine clinical practice, that APBI achieves survival outcomes comparable to WBI, with improved patient-reported quality of life and stable cosmetic results over extended follow-up. Taken together, these results reinforce that multicatheter APBI is not only a safe and effective alternative to WBI but also a patient-centered approach that minimizes toxicity and enhances long-term well-being.

In parallel, GEC-ESTRO developed consensus guidelines for patient selection. The 2010 recommendations categorized patients as suitable, cautionary, or unsuitable based on age (≥ 50 years), tumor size (≤ 3 cm), margin width (≥ 2 mm), and absence of lymphovascular invasion. The updated 2018 ESTRO-ACROP guidelines introduced a more individualized framework, incorporating molecular subtypes and expanding eligibility to include patients ≥ 40 years with margins ≥ 1 mm in favorable settings. They also endorsed various APBI modalities, including interstitial brachytherapy, 3D-CRT, IMRT, and IORT.

The Budapest trial randomized 258 low-risk patients to receive WBI or APBI, with 88 patients in the latter arm treated using multicatheter brachytherapy. After a median follow-up of 17 years, outcomes showed equivalent 20-year OS and DFS, with lower late toxicity and better cosmetic results in the APBI group [17].

The NSABP B-39/RTOG 0413 trial—the largest to date with 4,132 evaluable patients—did not demonstrate equivalence in ipsilateral breast tumor recurrence between APBI and WBI. However, the absolute 10-year difference was small (< 1.6%), and no significant differences were observed in DFS or OS. Importantly, grade ≥ 2 toxicity was more frequent in the WBI arm (66% vs. 54%) [12].

Our results agree with previous studies: no differences in local control or survival, but APBI showed better long-term outcomes for toxicity, cosmesis, and quality of life. Though radiologic changes were more common with APBI, they didn’t affect patient-reported outcomes. WBI used 3D-CRT, now often replaced by IMRT/VMAT, but multicatheter brachytherapy remains more precise, sparing key organs. APBI patients experienced fewer acute skin toxicities, stable cosmetic results, and higher satisfaction, consistent with GEC-ESTRO and Budapest trials.

Quality of life, evaluated through validated tools (EORTC QLQ-BR23 and S-BIS), was significantly better in the APBI group. This supports prior evidence indicating improved body image and lower symptom burden with APBI [27]. Local recurrence rates in our cohort were also in line with GEC-ESTRO and Budapest data, and while NSABP B-39 noted a slight excess risk, this did not reach clinical relevance.

This is the longest follow-up study in Spain comparing prospectively recorded multicatheter APBI and WBI cohorts. Limitations include small sample size, few events, non-randomized design, and varied WBI regimens, especially with increased hypofractionation after 2010.

## Conclusions

These findings support multicatheter APBI as a safe, effective, and patient-friendly alternative to WBI. It should be considered a standard option for selected patients, offering less toxicity, better cosmesis, and quality of life without compromising cancer control.

## Data Availability

Data available under reasonable request to the corresponding author.
